# Risk Factors for Hospitalized Seasonal Influenza in Rural Western Kenya

**DOI:** 10.1371/journal.pone.0020111

**Published:** 2011-05-26

**Authors:** Maurice O. Ope, Mark A. Katz, Barrack Aura, Stella Gikunju, M. Kariuki Njenga, Zipporah Ng'ang'a, John Vulule, Robert F. Breiman, Daniel R. Feikin

**Affiliations:** 1 Department of Disease Surveillance and Response, Ministry of Public Health and Sanitation, Nairobi, Kenya; 2 Field Epidemiology and Laboratory Management Training Program, Centers for Disease Control and Prevention, Nairobi, Kenya; 3 Jomo Kenyatta University of Agriculture and Technology, Nairobi, Kenya; 4 Influenza Program, Global Disease Detection Division, Centers for Disease Control and Prevention, Nairobi, Kenya; 5 International Emerging Infections Program, Global Disease Detection, Centers for Disease Control and Prevention, Nairobi, Kenya; 6 Center for Global Health Research, Kenya Medical Research Institute, Kisumu, Kenya; University of Hong Kong, Hong Kong

## Abstract

**Background:**

Risk factors for influenza hospitalization in Africa are unknown, including the role of HIV.

**Methods:**

We conducted a case-control study of risk factors for hospitalized seasonal influenza among persons in rural western Kenya, a high HIV prevalence area, from March 2006- August 2008. Eligible cases were ≥five years old, admitted to health facilities with respiratory symptoms, and had nasopharyngeal/oropharyngeal swab specimens that tested positive for influenza A or B by real-time reverse transcription-PCR. Three randomly selected age-, sex- and neighborhood-matched controls were enrolled per case. A structured questionnaire was administered and home-based HIV testing was performed. Risk factors were evaluated using conditional logistic regression.

**Results:**

A total of 64 cases (38 with influenza A and 26 with influenza B) and 190 controls were enrolled. The median age was 16 years (range 5–69 years). Among cases, 24.5% were HIV-infected versus 12.5% of controls (p = 0.004). Among persons ≥18 years old, 13 (59%) of 22 tested cases were HIV-positive compared with 15 (24%) of 62 tested controls (p = 0.005). In multivariable analysis, HIV-infection was associated with hospitalization due to influenza [adjusted Odds Ratio (aOR) 3.56, 95% CI 1.25–10.1]. The mean CD4 count among HIV-infected cases and controls was similar (399 vs. 387, respectively, p = 0.89). Chronic lung disease (aOR 6.83, 95% CI 1.37–34.0) was also associated with influenza hospitalization in multivariable analysis. Active pulmonary tuberculosis was associated with influenza hospitalization in bivariate, but not multivariable, analysis.

**Conclusions:**

People with HIV infection and chronic lung disease were at increased risk of hospitalized influenza in rural Kenya. HIV infection is common in many parts of sub-Saharan Africa. Influenza vaccine might prevent severe influenza in these risk groups.

## Introduction

Despite the worldwide impact of influenza, to date most epidemiologic data on the burden of influenza have come from developed countries, with little data from sub-Saharan Africa [1], [2]. Only one study from South African children has calculated an incidence of severe seasonal influenza infection and little is known about the seasonality of influenza in Africa, outside of South Africa and Madagascar [2], [3]. Moreover, there is little diagnostic testing for influenza in clinical settings and rare availability of influenza vaccine, entirely in the private market, in most of Africa [2], [4]. Lastly, little is known about the risk factors for influenza infection in Africa [2].

There are several factors in Africa that might lead to unique epidemiologic characteristics and a different clinical spectrum of influenza disease. Comorbid infections, such as HIV, tuberculosis and malnutrition, are highly prevalent in Africa [2], [3], [5], [6]. In many parts of Africa, like western Kenya, over 15% of adults are HIV-infected and a third of children are malnourished (KEMRI/CDC unpublished data) [7], [8]. In addition, social mixing patterns are different than in developing countries, with more crowding within households and public places, like markets, which can lead to more opportunities for person-to-person transmission of influenza virus. As such, it is relevant to define distinct risk factors in the African setting for use in targeting prevention measures [2].

To better characterize the disease burden and epidemiology of seasonal influenza in Africa, population-based surveillance for hospitalized influenza was implemented in inpatient health facilities in Bondo District in rural western Kenya in 2006–2008. We conducted a retrospective, matched case-control study to identify risk factors, including HIV infection, for hospitalized influenza among persons ≥five years old.

## Methods

### Ethical review

This study was approved by the institutional review boards of US Centers for Disease Control and Prevention (CDC) and Kenya Medical Research Institute (KEMRI). Written informed consent was obtained for all participants for questionnaires and HIV-testing, and for linking the two databases. For children aged 5–6 years old, parental consent was obtained. For persons aged 7–17 years old, parental consent and individual assent was obtained.

### Setting

Bondo District, part of Nyanza Province, in rural, western Kenya, with approximately 238,078 residents in 1999, is predominantly of Luo ethnicity [9]. Fishing and subsistence farming are the main economic activities. Those rearing domestic animals commonly go to crowded animal markets to buy or sell animals. HIV prevalence in Nyanza Province is the highest in Kenya, 15.3% among 15–49 year olds in 2007 compared to the national average of 7.4%. [8]. The district, one of the poorest in Kenya, is holoendemic for malaria, and has a high child mortality rate [9]. Bondo District has seven (four public and three private) inpatient facilities, including one government-operated district hospital, and 45 outpatient facilities. Influenza vaccination was not available in Bondo during the study period.

CDC's Global Disease Detection Division, along with KEMRI, established surveillance for influenza in all seven inpatient facilities in Bondo District by May 2007. The Bondo surveillance was initiated as a population-based surveillance site to complement sentinel influenza surveillance undertaken by the Kenya Ministry of Health in several provincial hospitals in Kenya to better characterize seasonal and epidemic influenza. For this study, screening criteria followed a suggested definition of severe acute respiratory illness proposed for influenza surveillance in developing countries, defined as a hospitalized patient ≥five years old with cough, shortness of breath, difficulty in breathing, or pleuritic chest pain and either oxygen saturation of <90% or axillary temperature ≥38°C [10]. Of note, surveillance nurses examined patients after they were admitted; the admitting clinicians did not apply or use the same case definition in their decision to admit patients.

### Laboratory

Nasopharyngeal and oropharyngeal swabs were collected by nurses and placed together into one cryotube containing one mL virus transport media without antibiotics. Specimens from three hospitals were kept in a cool box with ice packs at 2–8°C that was transported daily to the KEMRI/CDC laboratory and then stored at −70°C until testing. In four facilities where daily transportation was not possible, specimens were stored immediately in liquid nitrogen for up to 2 weeks. The frozen specimens from all sites were transported to the testing laboratory on dry ice. Testing for influenza viruses was carried out after one freeze-thaw cycle using real-time reverse transcription-polymerase chain reaction (rRT-PCR). Samples were aliquoted and total RNA was extracted from 100 µL aliquots of each sample using Qiagen's QIAamp viral RNA minikit (Qiagen inc, Valencia CA, USA), according to the manufacturer's instructions. One step rRT-PCR was carried out using the AgPath kit (Applied Biosystems, California USA) and results obtained following procedures provided by the Centers for Disease Control and Prevention , USA (Lindstrom, personal communication). This protocol is available under a material transfer agreement from CDC upon request. Cycle threshold (CT) values ≤39.9 were considered positive. All results were reviewed by the laboratory supervisor before being considered final.

The standard Kenya Ministry of Health HIV testing algorithm was administered, using parallel rapid test kits using whole blood obtained from finger-prick -Determine HIV-1/2 (Abbott Diagnostic Division, Hoofdorp, Netherlands) and Bioline®(Standard Diagnostics Inc, Korea)-with tie-breaking of discordant results using Unigold™ (Trinitiy Biotech PLC, Ireland). Blood specimens for CD4 cell count were collected in EDTA-containing vacutainer tubes and transported at 20–25°C to the CDC HIV reference laboratory within 12 hours of collection. CD4 cell count was processed using a BD FACSCount™ machine (Becton-Dickinson, Maryland).

### Case-control study

We conducted an age-, sex- and neighborhood-matched case control study. Eligible cases were hospitalized persons who met screening criteria and had influenza A or B detected by rRT-PCR between 26^th^ March 2006 and 28^th^ August 2008. Case enrollment took place in their homes, several weeks to months after their hospitalization, due to delays between collection and testing of specimens. Three attempts were made to locate eligible cases. Three controls were sought for each case. Controls were matched using the following age strata; 5–9, 10–14, 15–19, 20–29, 30–39, 40–49, 50–59, 60–69 and ≥70 years. Persons who had a respiratory illness requiring hospitalization during the last one year were excluded as controls to prevent misclassification bias.

To identify the first eligible person to serve as a control, we spun a bottle at the gate of the case's house and then walked in the direction the bottle pointed. After passing the first homestead in that direction, we approached the second homestead, where we looked for an age-eligible person to serve as a control, including those not at home. If at home, we offered enrollment to the age-eligible person. If more than one age-eligible person lived in the household, we assigned them numbers. A number was randomly selected and that person was offered enrollment. If the identified age-eligible person was not home, at least three return visits were made to enroll them. If no controls were able to be enrolled in the identified homestead, we continued walking in the same direction until we found a homestead with an eligible person who agreed to be enrolled as a control. The second and third controls were selected in the next two homesteads in the same direction as the original bottle spin using the same methodology. A desired sample size of 53 cases and 159 controls was calculated using the expectation that 35% of cases would have HIV, the main risk factor of interest, compared with 15% of controls (the HIV prevalence among adults in Nyanza Province), using a power of 80% and a type I error rate of 5% [8].

A standardized, closed-ended questionnaire was administered to cases and controls by trained field workers. Cases were asked about exposures during the seven-day period prior to illness onset. Controls were asked about exposures during the same seven-day period as their matched case. Since interviews occurred up to three months after the time period in question, in order to aid people's memories during interviews, we mentioned notable local or national events that occurred during the reference period. Next of kin were interviewed for case-patients that had died. The risk factors assessed among both cases and controls included exposure to indoor smoke, smoking cigarettes, drinking alcohol, exposure to children, and presence of chronic medical conditions. The chronic medical conditions assessed included asthma, chronic heart and lung disease, diabetes and current pulmonary tuberculosis, all of which were identified by self-report and verified by a clinician's diagnosis or relevant medications that a clinician had prescribed, which were available from documentation in medical record books and prescription notes that the participant kept in the home. Chronic lung disease was defined as a history of prolonged or recurrent cough or chest pain lasting more than two weeks in a person without known asthma or pulmonary tuberculosis. We also asked about domestic animal ownership (i.e. cows, sheep, donkeys, and chickens) and number of animals owned.). Data on level of education (none, primary, secondary, post-secondary), household possessions (radio, sofa, lantern, TV, bicycle), and main source of income for the household head and spouse were also collected and used as proxy measures of socioeconomic status. Scannable paper forms were used (TeleForm® software , Cardiff™, Vista, CA). Inconsistent or illogical data were returned to the field for correction.

Home-based HIV testing was attempted on all cases and controls by a separate team of trained counselors. HIV-testing was done as described above.

### Analysis

Data were analyzed using EPI-INFO (version 3.4.1, Atlanta, GA) and SAS (version 9.2, Cary, NC). Bivariate analysis was performed, accounting for matching, producing a matched odds ratio with 95% confidence intervals using the Mantel-Haenzel method. P values were calculated for categorical variables using Mantel-Haenzel chi- square test or Fisher's exact test as appropriate, and the Wilcoxon rank sum test was used to compare continuous variables. Variables that had a p value <0.10 were included in conditional logistic regression. Using stepwise backward elimination, variables were removed until only variables with a p value <0.05 were retained. Interaction was assessed by inclusion of product terms for all variables remaining in the final model. Population attributable risk percent was calculated using the formula for case-control studies proposed by Bruzzi et al [11].

## Results

A total of 64 cases and 190 controls were enrolled. For two cases, only two matching controls could be found. Among cases, 38 (59.4%) were infected with influenza A virus and 26 (40.6%) with influenza B virus. The median age was 16 years (range 5–69 years); there was no statistically significant age difference between influenza A and influenza B patients (median 19.5 versus 14.5 years, p = 0.22). The subtypes for seasonal influenza A viruses from 17 cases in which sub-typing was done were H3N2 (ten cases) and H1N1 (seven cases).

The demographic characteristics of cases and controls were similar (table 1). A total of 53 (82.8%) cases and 136 (71.6%) controls were tested for HIV among the 57 cases and 173 controls approached for HIV testing. Participants were not tested for HIV either because they refused testing (n = 22) or migrated out of the area (n = 17) or had died (n = 2). There was no significant difference in age or sex of those tested compared with those not tested for HIV. Thirteen (24.5%) cases and 17 (12.5%) controls tested positive for HIV (p = 0.004). Among persons ≥18 years old, 13 (59%) of 22 tested cases were HIV-positive compared with 15 (24%) of 62 tested controls (p = 0.005). HIV-positive cases and controls did not differ in CD4 count or treatment status (Table 2). The principal admitting diagnoses given by the admitting clinicians among cases were malaria (29.0%), pneumonia (27.4%) and diarrheal illness (4.8%); there were no differences in admitting diagnosis between HIV-positive and HIV-negative patients. The median number of days of hospitalization was two days (range zero to six); there was no significant difference in the median length of stay for HIV-positive and HIV-negative patients (2.0 vs. 1.5 days, p = 0.45). No case-patients died in hospital, but two (3.1%) died between hospital discharge and enrollment.

**Table 1:** Description of the study participants in case-control study of risk factors for hospitalized influenza, Bondo District, Kenya, 2007-9.

Factor	Cases, N = 64	Controls, N = 190	P value
Median age years (range)	16 (15–67)	16 (5–69)	0.70
Age in years-n (%)			
5–9	16 (25.0)	54 (28.4)	0.37
10–17	18 (28.1)	48 (25.3)	
18–34	12 (18.8)	51 (26.8)	
35–49	13 (20.3)	22 (11.6)	
≥50	5 (7.8)	15 (7.9)	
Male–n (%)	30 (46.9)	89 (46.8)	1.0
Education–n (%)			
None	9 (14.1)	30 (15.8)	0.91
Primary	50 (78.1)	142 (74.7)	
Secondary and above	5 (7.9)	18 (9.5)	
Mean household size (SD)	5.9 (2.5)	5.6 (2.5)	0.50
Household with children<5 years–n (%)	43 (67.2)	120 (63.2)	0.54
Keep domestic animals–n (%)	64 (100)	182 (95.8)	0.09
Use firewood for cooking–n (%)	57 (89.1)	170 (89.5)	0.93
Cooking inside the house–n (%)	35 (59.3)	86 (55.1)	0.58
Smokes cigarettes–n (%)	3 (4.7)	7 (3.7)	0.72
Drinks alcohol–n (%)	3 (4.7)	15 (7.9)	0.29

**Table 2:** Selected treatment characteristics of HIV-positive participants in case-control study of risk factors for hospitalized influenza, Bondo District, Kenya, 2007-9.

Characteristic	Cases N = 13 n (%)	Controls N = 17 n (%)	P value
On antiretroviral therapy	3 (23.1)	3 (17.6)	0.53
On Septrin prophylaxis	2 (15.4%)	4 (23.5%)	0.47
CD4 cell count (mean±SD)^a^	399 (184)	387 (234)	0.89
CD4 count^a^			
<250	1 (10.0%)	5 (33.3%)	0.29
250–499	7 (70.0%)	6 (40.0%)	
≥500	2 (20.0%)	4 (26.7%)	

a Blood was tested for CD4 cell count for 10 cases and 15 controls only.

In bivariate analysis, several chronic underlying illnesses, including chronic lung disease, current pulmonary tuberculosis and HIV infection, were associated with influenza A and B hospitalization (Tables 3, 4, 5). Ownership of cattle and chickens were also associated with influenza hospitalization. Exposure to indoor smoke, smoking cigarettes, educational status, source of income and socioeconomic status, as determined by household possessions, were not associated with influenza hospitalization. Although those who had co-infection of HIV and pulmonary tuberculosis (n = 3) were at high risk of hospitalized influenza, this association did not achieve statistical significance (OR_MH_ 6.00, 95% CI 0.54-66.17).

In multivariable analysis of influenza hospitalization (either A or B), chronic lung disease, which excluded active pulmonary TB and asthma, had the strongest association with influenza hospitalization (aOR 6.83, 95% CI 1.37–34), although only 6 (9.4%) cases had chronic lung disease, yielding a population attributable risk percent of 8% (Table 3). HIV-infected persons were 3.56 times more likely to be hospitalized with influenza (95% CI 1.25–10.07). The population attributable risk percent of HIV infection was 15%. Patients with influenza hospitalization were two times more likely to own cattle than controls (aOR 2.02, 95% CI 1.01-4.07). Also, the risk of influenza hospitalization increased by 4% (95% CI 1–7%) with each additional chicken owned.

**Table 3:** Results of bivariate and multivariable analysis of risk factors for hospitalized influenza A and B, indicating the factors that were included in the multivariate model (p<0.10), Bondo District, Kenya, 2007-9.

			Bivariate		Multivariable
Risk factor	Cases N = 64 n (%)	Controls N = 190 (%) n (%)	OR_MH_ (95% CI)	P value	Adjusted OR (95% CI)
Current Pulmonary TB	4 (6.3)	1 (0.5)	12.00 (1.34–107.37)	0.0045	NS
Chronic lung Disease	6 (9.4)	3 (1.6)	5.89 (1.47–23.65)	0.005	6.83 (1.37–33.98)
Chronic Heart Disease	3 ( 4.7)	1 (0.5)	9.00 (0.94–86.53)	0.021	NS
HIV positive^a^	13 (24.5)	17 (12.5)	7.00 (1.69–28.93)	0.0041	3.56 (1.26–10.07)
HIV status unknown	11 (21.6)	54 (31.2)	0.56 (0.25–1.26)	0.15	0.82 (0.37–1.85)
Owns cattle	44 (68.8)	101 (53.2)	2.10 (1.09–4.04)	0.024	2.02 (1.01–4.07)
Median number cattle (range)	3 (0–40)	1(0–38)	--	0.013	NS
Median number of chickens (range)	9.5 (0–99)	7 (0–50)	--	0.022	1.04 (1.01–1.07)

a Total number of cases and controls tested was 53 and 136 respectively. Reference group is HIV-negative.

We assessed variables associated with influenza A hospitalization separately (Table 4). In multivariable analysis, HIV infection had the strongest association (aOR 3.87, 95% CI 1.13 – 13.23.) Hospitalized influenza A patients also had a higher median number of chickens than controls (aOR 1.05, 95% CI 1.01–1.08).

**Table 4:** Results of bivariate and multivariable analysis of risk factors for hospitalized influenza A, indicating the factors that were included in the multivariate model (p<0.10), Bondo District, Kenya, 2007-9.

			Bivariate		Multivariable
Risk factor	Cases N = 38 n (%)	Controls N = 114 (%) n (%)	OR_MH_ (95% CI)	P value	Adjusted OR (95% CI)
Current Pulmonary TB	2 (5.3%)	1 (0.9%)	6.00 (0.54–66.17)	0.096	NS
Chronic lung disease	1 (2.6%)	1 (0.9%)	3.00 (0.19–47.97)	0.44	NS
Chronic Heart Disease	2 (5.3%)	1 (0.9%)	6.00 (0.54–66.17)	0.096	NS
HIV positive^a^	8 (21.1%)	10 (8.8%)	4.70 (1.08–20.50)	0.035	3.87 (1.13–13.23)
HIV status unknown	6 (15.8%)	32 (28.1%)	0.68 (0.23–2.02)	0.49	0.58 (0.20–1.63)
Median number cows (range)	4 (0–30)	1 (0–38)	--	0.055	NS
Median number of chickens	10 (0–99)	7 (0–50)	--	0.015	1.05 (1.01–1.08)

a Total number of cases and controls tested was 32 and 82 respectively. Reference group is HIV-negative.

Influenza B hospitalization was associated with chronic lung disease and cattle ownership in bivariate analysis. Chronic lung disease remained strongly associated with hospitalized influenza in the multivariable model (aOR 7.11, 95% CI 1.38–36.75, Table 5). Although HIV-infection was not statistically significantly associated with laboratory-confirmed influenza B hospitalization, it was more common among cases (aOR 3.26, 95% CI 0.43–39.7).

**Table 5:** Results of bivariate and multivariable analysis of risk factors for hospitalized influenza B, indicating the factors that were included in the multivariate model (p<0.10), Bondo District, Kenya, 2007-9.

			Bivariate		Multivariable
Risk factor	Cases N = 26 n (%)	Controls N = 76 (%) n (%)	OR_MH_ (95% CI)	P value	Adjusted OR (95% CI)
Current Pulmonary TB	2 (7.7%)	0	--	0.063	NS
Chronic lung Disease	5 (19.2%)	2 (2.6%)	7.33 (1.42–38.00)	0.0064	7.11 (1.38–36.75)
HIV positive^a^	5 (23.8%)	7 (13.0%)z	Undefined	0.16	
HIV unknown	5 (23.8%)	22 (31.9%)	0.49 (0.12–1.90)	0.30	NS
Owns cows	19 (73.1%)	41 (53.9%)	3.10 (0.94–10.20)	0.073	NS

a Total number of cases and controls tested was 21 and 53 respectively. Reference group is HIV-negative.

## Discussion

This is the first study to describe risk factors for seasonal influenza hospitalization among older children and adults in Africa [2]. Our findings suggest that persons with chronic underlying illnesses, particularly HIV and chronic lung disease, are at increased risk of influenza-associated hospitalization, and would be priority candidates for influenza vaccination. While these factors are not unique to Africa, they are more common in Africa and therefore may play a larger role in the epidemiology of severe influenza.

Few studies have investigated the risk of influenza hospitalizations in HIV-infected persons [2]. In South Africa, HIV-infected children with confirmed influenza had an estimated eight-fold increased risk of hospitalization than did HIV-negative children [3]. In the same population, HIV-infected children with influenza-associated pneumonia had a higher case-fatality ratio (8%) than did HIV-negative children (2%) [12], [13]. No data are available before this study on the contribution of HIV infection to influenza hospitalization in African adults [2]. In the U.S., one study showed that HIV-infected women had the highest influenza-attributable risk for severe cardiopulmonary events of any high risk group [14]. In an outbreak of influenza in a rehabilitation center in Italy, HIV-infected persons had an increased risk of influenza-like illness (ILI), as well as undefined complications [15]. In an outbreak in a substance abuse rehabilitation center in New York, there was no increased risk of ILI among HIV-infected residents, although 5% of them were hospitalized versus none of the HIV-negative ILI residents [16]. In our study, like previous studies, CD4 count was not associated with risk of hospitalized pneumonia among HIV-infected persons [15]–[17].

Whether the increase in influenza hospitalization among HIV-infected persons is due to an increased susceptibility to influenza virus infection, a greater chance of developing severe complications of influenza or a lower threshold of clinicians to hospitalize HIV-infected persons is not clear. HIV-infected persons do appear to have higher rates of severe influenza complications, hospitalization and death than HIV-negative people, based on findings from developed countries [14], [16]–[18]. This is also likely the case in Africa, where co-morbidities among HIV-infected persons, such as tuberculosis, are even more prevalent [5], [6]. It is also possible that HIV-infected persons with influenza are more likely to develop secondary bacterial pneumonia leading to their hospitalization, as suggested by an increase in suspected bacterial pneumonia among HIV-infected children hospitalized with influenza in South Africa [3], [12]. Another potential explanation is that clinicians who know that someone with respiratory illness is HIV-infected might be more likely to hospitalize that person or that persons with HIV-infection who develop influenza are more likely to seek care at hospitals. We believe that the latter is likely not a large contributor in rural western Kenya, where more than two-thirds of HIV-infected persons did not know their HIV status [8]. Lastly, it is also possible that HIV-infected persons are admitted for other concomitant HIV-related diseases rather than for influenza. Fewer than 30% of case-patients had an admitting diagnosis of pneumonia; however, the influenza testing results were not available to hospital clinicians, who make most diagnoses empirically without radiologic or laboratory data, including blood smears for malaria, which tends to be over-diagnosed in this setting [19].

Characterizing an increased risk of influenza complications among HIV-infected persons would be especially important in sub-Saharan Africa, where 22 million people are estimated to have HIV/AIDS [8], [20]. Several interventions may decrease the influenza burden in HIV-infected persons. Highly active antiretroviral therapy (HAART) has been shown to decrease the risk of influenza complications [17], [21]. Although most HIV-infected Africans who need HAART are still not receiving it, the percentage is increasing as efforts to expand HIV testing and implement widespread HIV treatment increase in Africa [8]. Second, several studies suggest that influenza vaccine is effective in HIV-infected persons who are not severely immunocompromised [16], [17], [22], [23]. The only randomized controlled trial designed to address efficacy of influenza vaccine in HIV-infected persons showed a 20% reduction in influenza-like illness and 100% efficacy against lab-confirmed influenza [23]. Currently, influenza vaccine is not available in the public sector in most of sub-Saharan Africa [2], [4].

We found persons with chronic lung disease were at increased risk of influenza hospitalization, which is consistent with previous studies done in the U.S. [24], [25]. Pulmonary TB also had a significant association with hospitalized influenza in bivariate, but not multivariable, analysis. As with HIV, because we focused on influenza among hospitalized patients, we were unable to distinguish whether persons with TB and chronic lung disease are more likely to become infected with seasonal influenza viruses, or whether once infected they are more likely to develop severe disease requiring hospitalization. In addition, healthy controls might have had fewer contacts with the healthcare system in the past year, therefore lacking the opportunity to be diagnosed with pulmonary TB or chronic lung disease that cases had, leading to a misclassification bias.

We found that influenza hospitalization was associated with ownership of cattle and chickens. This association most likely reflects confounding, as animal-to-human transmission of influenza viruses, although documented, is very rare, especially for seasonal influenza viruses [26]–[29]. What the potential confounders might be are not clear. People who own domestic animals may have more contact with other people in crowded settings, such as animal markets, increasing the opportunity for influenza transmission. There might also be selection bias in that animal owners who were eligible to be controls might have been less likely to be at home or take the time to participate in the study. Additionally, animal owners might represent a different category of persons, with greater social mobility or access to healthcare, more likely to go to hospitals when ill, making them more likely to be cases.

Several limitations might have affected our study. First, HIV status at the time study enrollment was assumed to be the status at the time of influenza infection. While this was likely the case, it is possible that a few people had sero-converted in the interval between influenza virus infection and study enrollment, although this misclassification should have been non-differential between cases and controls. In addition, two cases died between hospital discharge and study enrollment and could not be HIV-tested by the study team. Because they died, they had a higher probability of being HIV-infected, which might have led to an underestimation of the true risk of HIV. Second, inherent in the design of case-control studies is the possibility of recall bias. Cases might have remembered some exposures more than controls. Moreover, many months often elapsed between illness and interviews, so that exposures in the week prior to illness might not have been remembered well. Nonetheless, many factors we evaluated are unlikely to change over time, and both cases and controls were asked to recall exposures during time periods similarly distant. Third, not all hospitalized patients with respiratory illness met the screening criteria, as we used an established severe acute respiratory illness surveillance case definition, so that some inpatients with influenza were not included [10]. In particular, we did not include patients without documented fever or hypoxia. Therefore, it is possible that our findings are not generalizable to all influenza-associated hospitalizations.

This study underscores the role of underlying conditions, particularly HIV infection, in hospitalized seasonal influenza patients in Africa. Persons with such conditions should be prioritized for influenza vaccination and other preventive strategies. The effect of these conditions on the burden of severe influenza disease during the recent 2009 H1N1 pandemic needs further exploration.
